# Single-Baseline RTK Positioning Using Dual-Frequency GNSS Receivers Inside Smartphones

**DOI:** 10.3390/s19194302

**Published:** 2019-10-04

**Authors:** Paolo Dabove, Vincenzo Di Pietra

**Affiliations:** Department of Environmental, Land and Infrastructure Engineering (DIATI), Politecnico di Torino, 10129 Torino, Italy; paolo.dabove@polito.it

**Keywords:** smartphones, real-time positioning, NRTK, GNSS positioning, mass-market devices

## Abstract

Global Navigation Satellite System (GNSS) positioning is currently a common practice thanks to the development of mobile devices such as smartphones and tablets. The possibility to obtain raw GNSS measurements, such as pseudoranges and carrier-phase, from these instruments has opened new windows towards precise positioning using smart devices. This work aims to demonstrate the positioning performances in the case of a typical single-base Real-Time Kinematic (RTK) positioning while considering two different kinds of multi-frequency and multi-constellation master stations: a typical geodetic receiver and a smartphone device. The results have shown impressive performances in terms of precision in both cases: with a geodetic receiver as the master station, the reachable precisions are several mm for all 3D components while if a smartphone is used as the master station, the best results can be obtained considering the GPS+Galileo constellations, with a precision of about 2 cm both for 2D and Up components in the case of L1+L5 frequencies, or 3 cm for 2D components and 2 cm for the Up, in the case of an L1 frequency. Moreover, it has been demonstrated that it is not feasible to reach the phase ambiguities fixing: despite this, the precisions are still good and also the obtained 3D accuracies of positioning solutions are less than 1 m. So, it is possible to affirm that these results are very promising in the direction of cooperative positioning using smartphone devices.

## 1. Introduction

In the last few years, thanks to the available microtechnology, some significant advances have been made in the field of mass-market positioning. The Global Navigation Satellite System (GNSS) chipsets mounted on new smartphones generation, with their low production cost and the microelectronic components miniaturization, are leaving a footprint in an ever-growing consumer electronics market. This led to the spread of location-based applications and services (LBS), which the user can use to perform numerous spatial related tasks. Navigation, advertising, social networking, entertainment, and health are examples of the numerous LBS operational contexts. However, questions remain in the research field: how does the miniaturization of the sensors degrade their performances, and which is the level of positioning accuracy that is reachable with these devices?

The everyday evidence demonstrates the capability of these devices to perform positioning and navigation, with some meters also showing high accuracy in complex environments. Compared to more complex and expensive geodetic receivers, these smartphones’ System of Chipsets exploit external assistance information provided by alternative communication channels like the internet connection of a cellphone network. These techniques, called Assisted GNSS (A-GNSS) improve standard GNSS chipset performances and allow the chipsets to be used in a real-time fashion. In fact A-GNSS reduces the time required to perform the Time To First Fix [1] and improves the positioning accuracy, thereby providing a form of assistance composed by fast messages for ephemeris, almanacs, orbits, and corrections. Nowadays, under good multipath conditions, the typical accuracy reachable is about few meters while in adverse conditions it becomes around 10 meters. In general, the accuracy of the latest GNSS navigation messages is approximately 1 meter, making the main sources of errors the front-end antenna and the receiver-dependent noise. Geodetic receivers allow it to reach sub-centimetric positioning accuracy thanks to the quality of the geodetic antenna, its regular gain, the high sensitive oscillator, and the signal processor performances. Moreover, their capability to acquire multi constellation and multi-frequency observations allows it to apply advanced algorithms for positioning. Numerous techniques can be used in real-time and post-processing in order to estimate and compensate for the biases affecting the so called GNSS observables, pseudoranges, and carrier-phase measurements. The three main techniques for increasing the accuracy of the positioning are based on differentiation, modeling, and a combination of these measurements. While pseudoranges are mainly used for low-precision real-time positioning, carrier-phase measurements are used for precise positioning, both in real-time and in post-processing. 

Until recently, both mass-market receivers and chips installed in smartphones exploited mainly code-phase measurements on the single frequency L1/E1 despite having an internal carrier-phase loop. This was due to their inability to demodulate the carrier-phase signals and due to the need to operate in real-time. Although in the most modern receivers this is no longer a problem, the research on smartphone positioning initially focused on the feasibility of cm-accurate positioning based only on pseudoranges measurements and analyzing the performances of the main parts of the GNSS chipsets like the internal oscillator and the antenna gain. In fact, it is known that the former, namely the internal oscillator, is due to the occurrence of some time correlated gaps in the pseudorange measurements, while the latter leads to poor multipath suppression capability and less sensitivity with respect to a geodetic antenna. Being unable to access the raw observation measurements, the classical approach used to analyze these problems consisted of using a hardware add-on or modification with external tools. For example, reference [2] deviates the analog signal acquired by the inner smartphone antenna directly to an external radio frequency front-end and GNSS receiver. This approach enabled them to apply a well-defined signal processing algorithm, avoiding the limitation of the internal chipset [3]. This test revealed the antenna gain to be the primary impediment to reliable positioning solution with a decrease of 11 dB in the carrier to noise ratio (C/N_0_) with respect to a survey-grade antenna. Humphreys et al. [4] gave more details on the performances of smartphones for cm-accurate positioning. Firstly, they were able to extract raw GNSS observables thanks to a Broadcom customized software implemented in an Android test phone. Having access to both pseudorange and carrier-phase measurements, the authors performed a differential GNSS processing in a zero-baseline configuration. The observations acquired by a survey-grade antenna and re-irradiated to a smartphone within a RF enclosure allowed them to directly test the single frequency GNSS chipset in a single point positioning mode. The results showed an accuracy of less than 5 meters horizontally, a result coherent with the expected deviation of a survey-grade receiver [4]. Again, the multipath-induced phase errors from the antenna remained the main challenge for accurate smartphone positioning.

A fundamental push for mobile positioning research came in May 2016, when, during the I\O conference, Google announced the release of a new Android operating system (OS), the Nougat 7.0. In this new implementation, accompanied by the Application Programming Interface level 24, Google gave the ability to developers and researchers to directly access the GNSS measures without having to use custom smartphones and external devices [5]. Today this feature still provides great interest for developers and researchers, as it gives them the opportunity to develop advanced processing algorithms using pseudoranges, Doppler shifts, and carrier-phase observations.

Initially, the first empirical tests on these measurements were focused on the raw observables data evaluation. Zhang et al. [6] acquired raw static measurements with a Nexus 9 tablet in order to characterize the errors with respect to a reference solution performing the single difference approach considering only pseudoranges. They observed an error of around ± 20 meters, a pseudorange rate noise within ± 10 m/s, and a C/N_0_ average value of approximately 10 dB-Hz lower than the geodetic receiver used as the reference solution [6]. In the same configuration the carrier-phase rate and Doppler data have also been characterized with a value within ± 0.2 m/s. In Liu et al. [7], the zero-baseline approach has been applied for the noise characteristics of the GNSS observation of a smartphone.

At the same time, advanced post-processing algorithms have been applied with the aim of achieving centimeter positioning with smartphones [8,9]. To do this, the first step was the development of parsers capable of extracting the raw measurements in a format more suited to geodetic post-processing. Some developers have created converters that can produce a Radio Technical Commission for Maritime Services (RTCM) or Receiver-Independent Exchange (RINEX) formats files that are useful for use in complex software (GEO++ RINEX logger and rinexON). Thanks to these applications, numerous research has been made to increment the positioning performances exploiting multi-constellation observations, real-time, and post-processing estimation procedures and signal processing. In reference [10], the authors were able to reach sub-meter accuracies with an on-board Doppler filtering algorithm and while considering the Satellite-based Augmentation Systems (SBAS). In reference [11], a decimeter level of accuracy in terms of positioning performances has been achieved following the post-processing approach, made by double differencing raw smartphone observations with those coming from a GNSS Continuously Operating Reference Stations (CORS) network. Also the present authors in reference [12] performed a Network Real Time Kinematic (NRTK) positioning to demonstrate the possibility of obtaining a centimeter-level accuracy through the use of differential corrections provided by CORS networks, even if it was not possible to fix the phase ambiguities in a correct way. Despite this challenge, sub-meter accuracy was obtained using that type of technique.

Another important milestone in smartphone positioning and navigation was in September 2017, when a chip manufacturer company, Broadcom Limited, announced the release of the first dual-frequency GNSS chipset, the BCM47755. Up to 2017, most GNSS chipsets installed inside smartphones were single-frequency receivers able to only provide measurements related to one frequency (the L1 band). In these cases, it is not possible to apply the double or triple differences approach [13,14], or to combine different observations [15]. The ability to use dual-frequencies signals, however, enables a higher level of positioning performances, mainly increasing the signal robustness to environmental impairments and the positioning accuracy.

The release of the first dual-frequency chipset, the first smartphone equipped with dual-frequency GNSS appeared in early June 2018, which was the Xiaomi Mi 8. The availability of signals from two frequencies allows us to correct most of the error sources introduced by ionospheric propagation. At the same time, it opens the way to solving the carrier phase integer ambiguity, enabling Real-Time Kinematic (RTK) and Precise Point Positioning (PPP) algorithms directly on smartphones, as long as the phase measurements are stable; furthermore, the design of the signals in the E5/L5 frequency makes it easier to distinguish real signals from the ones reflected by buildings, reducing the multipath effect [16]. Finally, frequency diversity is among the most regularly quoted solutions to increase robustness to interference and jamming. In addition to such benefits, the enhanced position precision also creates interesting opportunities for novel applications, such as augmented reality, autonomous vehicle navigation, and mapping.

The first research on this GNSS chipset has been made by reference [17] which evaluates the performances of the Xiaomi Mi 8 comparing the L5/E5a and L1/E1 frequencies. Applying double differences on GPS L5 and L1 code observations in a short baseline configuration, the precision of the measurements is 1.3 meters and 2.1 meters, respectively. Carrier phase-based static differential positioning using GPS and Galileo on a very short baseline has provided cm-level precision in the horizontal component and decimeter-level in the vertical component. In reference [18], the dual-frequency GNSS chipset mounted on the Xiaomi Mi 8 has been considered for multipath characterization, comparing the obtained results with those obtainable with a geodetic receiver. The comparison between GPS and Galileo measurements showed a lower multipath error in the last one, although these results are still very far from the values of the geodetic receiver. Again, the antenna represents the main limitation to achieving cm-level accuracy in positioning.

Starting from the previous work made by the authors of reference [12] on single frequency smartphone receiver, in which the RTK positioning has been performed using the differential corrections provided by the CORS network; the main aim of this work is to perform a relative positioning using two different Xiaomi Mi8 smartphones with a double-frequency GNSS chipset embedded. When using a short baseline configuration (less than few kilometers), it is possible to assume that common spatial-related biases affect both devices; thus, the goal is to show the positioning performances of a smartphone in case of static surveys with short baselines, if the master station is a smartphone as the rover station.

## 2. Smartphones Dual Frequency GNSS Receivers

**Figure 1:**
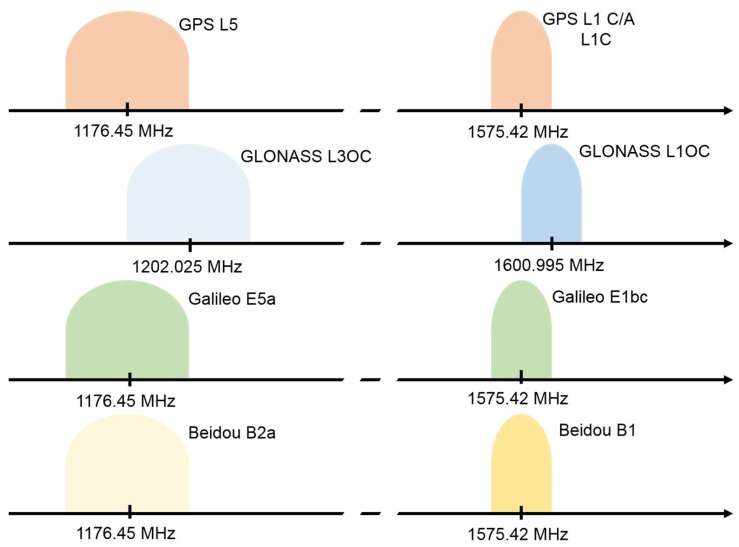
Global Navigation Satellite System (GNSS) signal structures considering only bands around GPS L1 and L5 frequencies, which were used in these tests.

As previous stated, until 2018, smartphones multi-constellation GNSS chipsets were able to acquire only one frequency signal from each satellite of the different constellations (GPS L1, Galileo E1, BeiDou B1, and GLONASS L1). In June 2018 the Xiaomi MI 8 smartphone was introduced to the market: this smartphone is the first device equipped with the Broadcom BCM47755 GNSS chipset, the first dual-frequency mass-market receiver designed by the Broadcom Limited company (San Jose, CA, USA). In addition to the previously described frequencies, this chipset uses also the GPS L5 and the Galileo E5a frequencies (Figure 1). This feature is an important step forward in the positioning field and in particular for real-time smartphone-based navigation.

Acquiring observations on more than one frequency allows us to perform the data combination procedure which uses GNSS observations acquired by the same receiver at the same station in order to estimate GNSS biases. The new dual-frequency receiver is able to acquire both code and carrier-phase measurements, which means that the combination can be made on both these observables.

In the state of art, the first and most important application of dual-frequency combination methods is the estimation and elimination of the ionospheric effects due to their dependency on the signal frequency. The ionospheric effect is an important error source in GNSS positioning as it generates a delay in the order of tens of meters. Combining code and carrier-phase observations linearly, it is possible to eliminate the ionospheric effect in the measurements. This procedure is called Iono-free combination. Another combination method is the geometry-free code-code or phase-phase combination, which removes all the errors in the observation equations except for the ionospheric term and the ambiguity parameters. Other possible linear combinations of carrier-phase measurements are the so called wide-line (difference) and narrow-line (sum) combinations.

Finally, frequency diversity could increase signal robustness to interference and jamming together with increasing the capability to distinguish between the real line of sight signals from the reflected ones.

## 3. RTK Positioning Using Smartphones

Common trends and research in the domain of centimetric RTK positioning have been already been presented in the introduction and outline the possibility of using carrier-phase measurements to perform RTK smartphone positioning and navigation with the low-grade smartphone antenna (the main error source) and power saving option of the Android OS. Dabove and Di Pietra [12] have demonstrated the possibility to obtain decimetre-level accuracy in smartphone NRTK positioning by processing undifferenced and uncombined observations from a single frequency GNSS chipset. The requirement in this research was to apply differential corrections provided by a GNSS CORS network for a smartphone GNSS receiver in real-time.

On the other hand, the objective of the present work is to address the problem of RTK positioning using smartphones in situations where the differential corrections are not available for many reasons (e.g.; because there is no internet connection or there are no CORS networks). For geodetic surveying, this problem can be addressed using the traditional single-base master-rover configuration of two GNSS receivers: as described in references [13,19], generally one master station (indicated with subscript A in the Equations (1) and (2)) is settled on a known point that can broadcast pseudorange (PRC, Equation (1)) or carrier-phase corrections (CPC, Equation (2)) to a rover receiver through internet connection or radio modem, if pseudorange and carrier-phase measurements are considered, respectively.

(1) $$PRC^{p}(t)=\rho_{A}^{p}(t)-R_{A}^{p}(t)-cdt^{p}(t)-cdT_{A}(t)=\alpha I_{A}^{p}(t)-T_{A}^{p}(t)-E_{A}^{p}(t)$$

(2) $$CPC(t)=\rho_{A}^{p}(t)-\phi_{A}^{p}(t)-\lambda N_{A}^{p}-cdt^{p}(t)-cdT_{A}(t)=-\alpha I_{A}^{p}(t)-T_{A}^{p}(t)-E_{A}^{p}(t)$$

In equations (1) and (2) $\rho_{A}^{p}$ represents the geometric range, $R_{A}^{p}(t)$ and $\phi_{A}^{p}(t)$ the pseudorange, and carrier-phase measurements, respectively, $cdT_{k}$ and $cdt^{p}$ are the biases related to receiver and satellite clocks multiplied by the speed of light, $\alpha_{i}I_{k}^{p}$ is the ionospheric propagation delay with a known coefficient $\alpha_{i}=f_{1}^{2}/f_{i}^{2}$ that depends on the i-th frequency, $T_{k}^{p}$ represents the tropospheric propagation delay, $Mi_{k}^{p}$ and $E_{k}^{p}$ the multipath and the ephemeris error, and finally $\lambda_{i}Ni_{k}^{p}$ the carrier-phase ambiguity multiplied by the wavelength, under the assumption that the random errors $\varepsilon_{k}^{p}$ are not shown in these equations.

After these estimations, the master station can broadcast the PRC and CPC values to the rover receiver, (defined as B) which in turn can exploit them through Equations (3) and (4).

(3) $$R_{B}^{p}{\left(t\right)}_{correct}=R_{B}^{p}(t)+PRC(t)=\rho_{B}^{p}(t)-cdT_{AB}(t)+\Delta E_{AB}^{p}(t)-\Delta I_{AB}^{p}(t)+\Delta T_{AB}^{p}(t)$$

(4) $$\varphi_{B}^{p}{\left(t\right)}_{correct}=\rho_{B}^{p}(t)+CPC(t)=\rho_{B}^{p}(t)-cdT_{AB}^{}(t)-\lambda N_{AB}^{p}+\Delta I_{AB}^{p}(t)+\Delta T_{AB}^{p}(t)+\Delta E_{AB}^{p}(t)$$

The subscript AB means that the bias is referred to the master-rover combination.

Considering both geodetic and mass-market GNSS receivers [20], if the distance between master and rover receivers is lower than 10 km, it is possible to affirm that the propagation of atmospheric delays and the ephemeris errors is almost the same in both places [21]. Thus, it is possible to eliminate them by differencing measurements of the two receivers: in the case of using carrier-phase measurements, it is possible to reach a centimetric level of accuracy if the phase ambiguity $N_{AB}^{p}$ is estimated (or fixed) as an integer number [11,22,23,24,25].

Starting from these considerations, the present work investigates two different situations of real-time positioning: firstly, it is interesting to verify the accuracy and precision of a smartphone device used as rover if a CORS is used as a master. Then, this master station is replaced with a smartphone receiver, while always considering a smartphone as the rover.

## 4. Test Setup

As discussed in the Introduction, one of the problems encountered using smartphone GNSS devices is to be able to know if the GNSS measurements are pre-filtered from the internal chipset. For this reason, a specific tool has been developed by the authors in order to get raw measurements directly from the device without any other pre-filtering operation. A dedicated code developed by the authors has been considered for processing all data collected in real-time, both from the master and rover receivers, in order to perform the single-base RTK positioning. As described in the previous section, two different receivers have been settled on a known point as a master station: the first one is a multi-frequency and multi-constellation GNSS receiver (TORI permanent station) that is part of the EUREF permanent network (www.epncb.oma.be/). The second one is a multi-frequency and multi-constellation smartphone device (Xiaomi Mi8). The main characteristics of these devices are summarized in Table 1. The rover device considered in these tests has the same characteristics of the smartphone used as the master station: in this case, the rover receiver (about 1 km far from the master station) has been settled on a known point in order to compare the estimated results with the reference coordinates. These reference results have been obtained using a geodetic GNSS receiver settled for 12 hours on the same point and post-processing. These observations followed a network adjustment computed with the Bernese GPS 5.0 software in the same reference frame of the master stations: ETRF2000(2008.0) [26,27]. The GNSS positioning has been performed considering different sessions with a length of about 10 min during November and December 2018, repeated in different days and hours (in order to make all results have independent atmospheric conditions and satellite geometry distributions), and with a sampling rate of 1 Hz. The entire conditions around the smartphones have been considered, paying attention to avoid multipath effects, electromagnetic disturbances, and building obstructions.

## 5. Experimental Results

**Figure 2:**
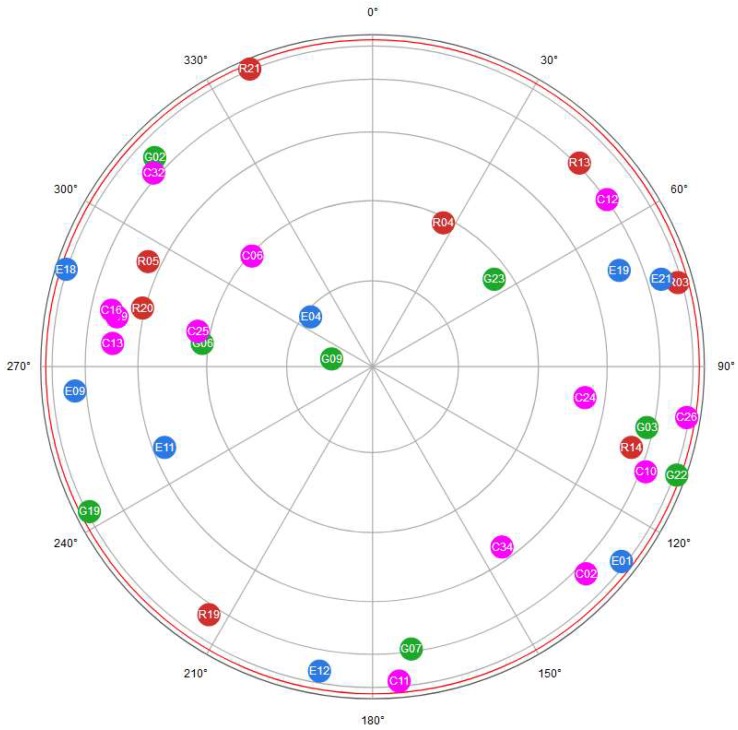
Skyplot of visible satellites in one of the selected epochs of measurements: green, red, blue and purple circles represent the GPS, GLONASS, Galileo, and BeiDou satellites, respectively.

In this section, the obtained results are discussed while considering the two tested configurations separately. Generally, for each epoch it was possible to track 8 GPS, 8 GLONASS, 6 Galileo, and 11 BeiDou satellites, respectively, as shown in Figure 2, obtaining a maximum GDOP value equal to 1.3. These values refer to the average of the different sessions.

**Figure 3:**
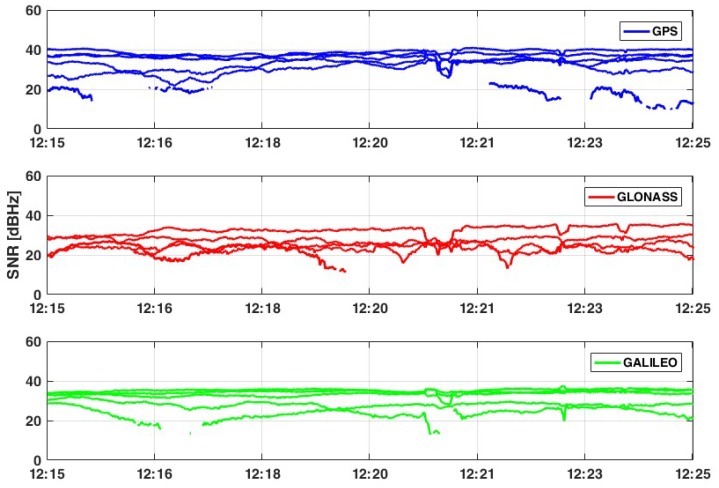
Signal to noise ratio (SNR)values for tracked satellites - L1 frequency.

**Figure 4:**
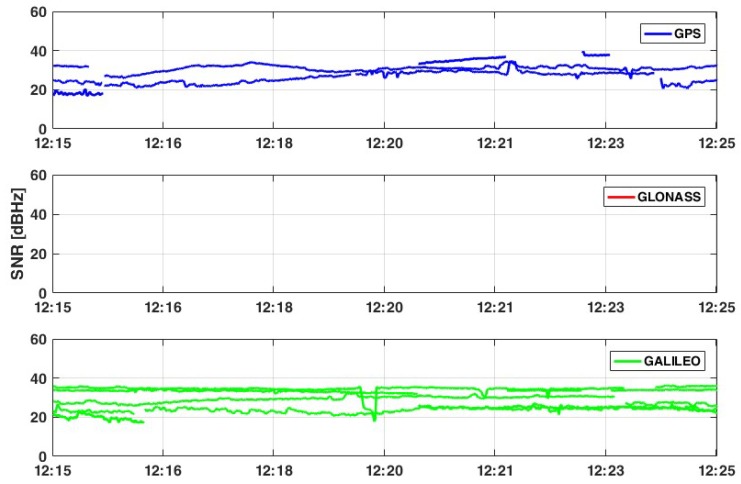
SNR values for tracked satellites - L5 frequency. No GLONASS satellites can track L5 frequency.

From a quality point of view of the signals, it is possible to note (Figure 3 and Figure 4) that in some cases, the quality is not good, even if the cutoff angle has been chosen to be equal to 10°. This happens when the signal to noise ratio (SNR) value is less than 25 dB-Hz, defining the satellite signal being as too noisy to be processed. In this context, only satellites with an SNR value greater than 28 dB-Hz and with an elevation greater than 15° are considered. Applying these filters, the number of available satellites decreases to 21: 7 GPS, 4 GLONASS, 3 Galileo, and 7 BeiDou.

According to Table 2 and considering only satellites that provides SNR values greater than 28 dB-Hz, it is possible to affirm that the noise of signals is still quite high, so the expected result could be noisy.

As previously written, two different scenarios have been considered (Table 3) whose results are reported separately. All data are processed considering a modified version of RTKLIB 2.4.3 [28], while only using the “continuous” method for the integer ambiguity resolution and a tight combination for multi-GNSS RTK positioning. As described in reference [12], “continuous” means that integer ambiguities are continuously estimated and resolved. Moreover, the “ratio test” for the standard integer ambiguity validation strategy has been considered. This factor, that can be considered also as a threshold, means the ratio of the squared sum of the residuals with the second best integer vector to the the best integer vector, as shown in (5).

(5) $${\left(\sigma_{0}^{2}\right)}_{2nd}/{\left(\sigma_{0}^{2}\right)}_{1st}\geq ratio$$

So, when the previous inequality (5) is satisfied, the ambiguities are defined as integer values, so it is possible to define that solution as “fix”, otherwise as “float”. As described in reference [12], a common value for the threshold is equal to 3, while it can be extended to 30 in order to guarantee a better and more feasible estimation.

Even if BeiDou satellites can be tracked by both receivers, the software used in these research activities is not able to process this constellation properly. Thus, we unfortunately are not able to provide results that also consider BeiDou satellites.

### 5.1. Case 1: Geodetic Master—Smartphone Rover

The first considered case is represented by a typical relative RTK positioning where the master station is composed by a geodetic multi-frequency and multi-constellation receiver (CORS station) settled on a well-known point and a rover device. This should represent the best possible solution for a relative positioning approach.

**Figure 5:**
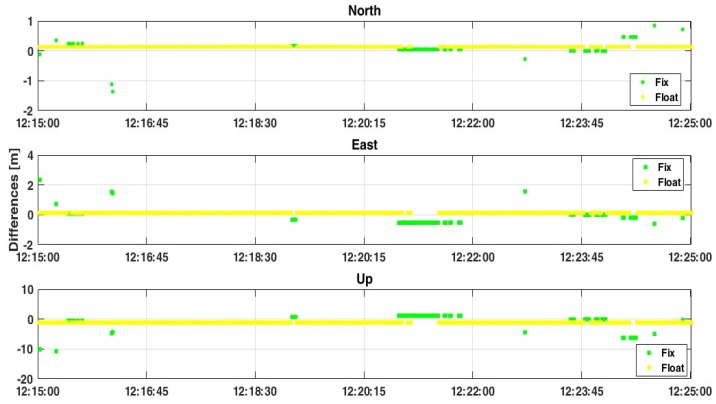
Positioning results considering case 1, with GPS-only constellation, L1 frequency, “continuous” method for the ambiguity resolution and ratio = 3.

Considering only the L1 frequency, GPS constellation, and “continuous” method for the ambiguity resolution, the results are quite good in terms of float solutions, even if 15% of epochs are estimated with phase ambiguities declared as “FIX” (where FIX means that they are estimated as integer values) but in a wrong way, as shown in Figure 5. As detailed in reference [12], the goal is not to reach a “FIX” solution, but it is preferable to guarantee the continuity and the quality (in terms of precision and accuracy) of the solutions without fixing the phase ambiguities as integer values. For this reason, all data have been processed again using a threshold for the ratio-test equal to 30: this means if the ratio of the squared sum of the residuals with the second best integer vector to with the best integer one is greater than this threshold value, the phase ambiguities are declared as “FIXED”, otherwise they are declared as “FLOAT”.

**Figure 6:**
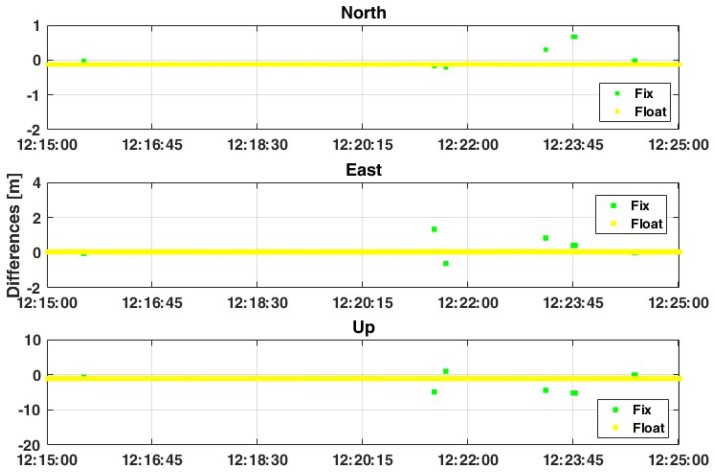
Positioning results considering case 1, with GPS-only constellation, L1+L5 frequencies, “continuous” method for the ambiguity resolution and ratio = 3.

As shown from Figure 6, the average values between estimated and reference coordinates are not substantially changed, even if there is a strong improvement in terms of the precision of solutions. As shown from Table 4 the standard deviation (std) values decrease from about 14 cm and 52 cm in the case of FIX+FLOAT solutions up to 0.2 cm and 0.5 cm in the FLOAT-only case, considering the 2D and Up components, respectively. This means that the software is trying to fix the phase ambiguities but the quality of carrier-phase measurements is too bad for reaching a good “FIX” solution, as described in reference [12]. Although it is quite common to set the ratio threshold to be equal to 3 [20], in this case the variability of carrier-phase measurements allows the software to reach the fixing of the phase ambiguities with 15% (Figure 5) of epochs but in a wrong way. The RMS values in the second case are slightly better than in case of FIX+FLOAT solutions, even if these values are still around 0.2–0.4 m and 0.5 m for North, East, and up components, respectively.

**Figure 7:**
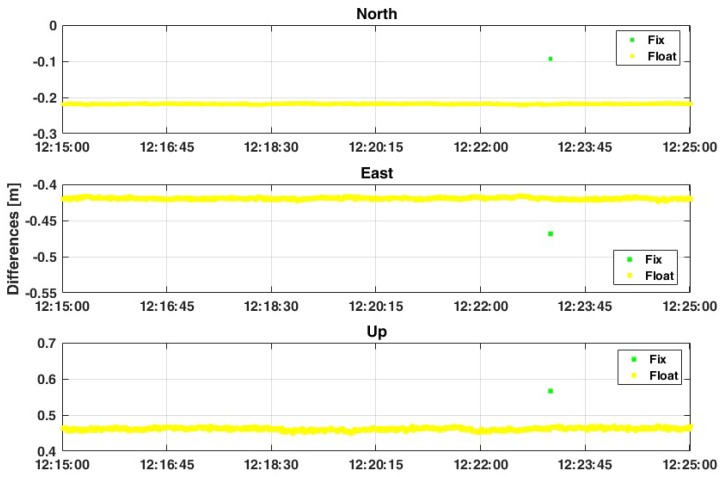
Positioning results considering case 1, with GPS-only constellations, L1+L5 frequencies, “continuous” method for the ambiguity resolution and ratio = 30.

If the second frequency (L5) is considered, the results are definitely better: the percentage of FIX solutions decreases by up to 0.6% (Figure 6) and an improvement in terms of precision and accuracy is observed. As shown in Table 5, the use of L5 frequency allows the increasing of the precision (std) with an order of magnitude, going from about 14 cm and 30 cm for planimetry and up components in case of L1-only solution up to 1.5 cm and 3.8 cm in case of L1+L5 approach. These results can be still improved if the threshold value of the ratio-test is increased up to 30 (Figure 7): Table 6 shows that the std values are closed to 0.1 cm and 0.4 cm for planimetry and up components in the case of FLOAT-only solutions, while they are about 1.5 cm and 3.8 cm in the case of the FIX+FLOAT approach. Also RMS values are decreased, even if the difference is not so high: in this case the main improvement is for the up component, where the RMS value is decreased by about 12 cm, from 59.6 cm to 47.6 cm.

The previous results, shown in Table 3 and Table 4.; are representative of those obtained in many other sessions that are not presented in this paper: thus, both mean and standard deviation values can be considered as reference values for this positioning technique, using these sensors.

### 5.2. Case 2: Smartphone Master—Smartphone Rover

**Figure 8:**
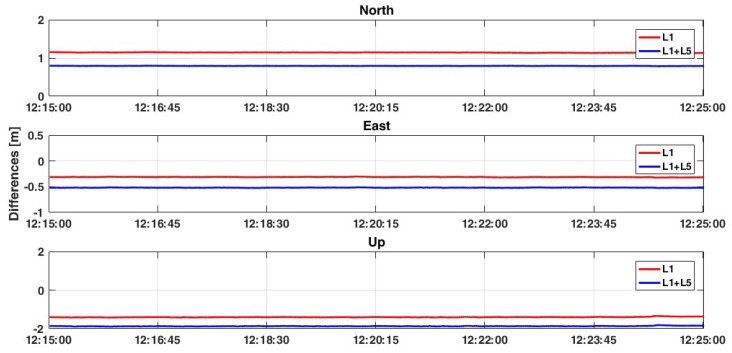
Comparison between L1 and L1+L5 solutions considering the GPS-only constellation and ratio = 30.

In this second case, the relative positioning is performed considering one smartphone as master device and another smartphone as rover. This represents an innovative approach where both the master and rover are mobile devices: this is a preliminary step towards cooperative positioning using GNSS signals extracted by smartphones. As was previously written, all data have been collected simultaneously with the geodetic receiver, in order to compare results considering the same satellites (signal quality and geometry distribution) and atmospheric conditions. Moreover, based on previous results, only FLOAT solutions have been analyzed. The obtained results are very promising, which are reported in Table 7, Table 8, Table 9 and Table 10. Considering the L1+L5 frequencies of GPS constellation, it is possible to observe 2D positioning accuracy at less than 1 meter and accuracy of the Up component at less than 2 meters (Figure 8). Moreover, although the accuracy is not increased with respect to the single frequency solution, the standard deviations decrease to about 1 cm. Also, the RMS values are decreased, with a high improvement especially for the north and up components (from 1.147 m and 1.386 m up to 0.230 m and 0.463 m, respectively).

**Figure 9:**
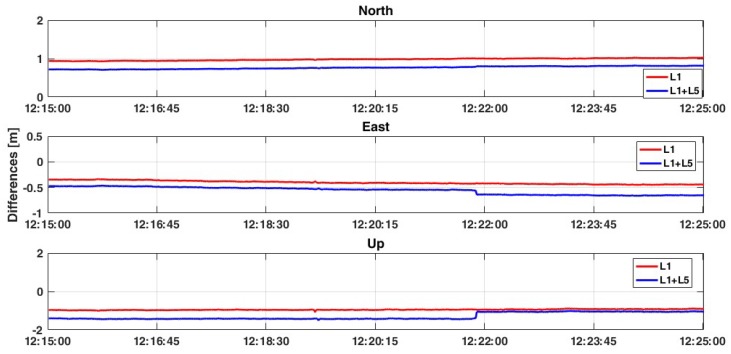
Comparison between L1 and L1+L5 solutions considering GPS+GLONASS constellations with a ratio = 30.

When GLONASS measurements are also considered, a slightly different behaviour is observed. Although the positioning solution is similar to the GPS-only, both the accuracy and the precision decrease when dual-constellation measurements are considered (Figure 9). This means that GLONASS measurements introduce some noise in the positioning estimation. Also, RMS values are slightly worse, even if no substantial differences can be observed with respect to the previous case.

**Figure 10:**
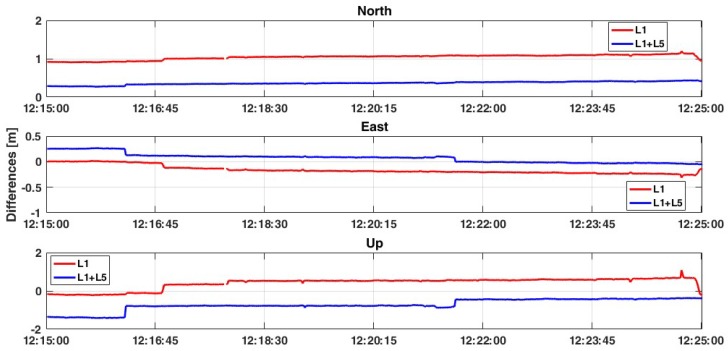
Comparison between L1 and L1+L5 solutions considering GPS+GLONASS+Galileo constellations with a ratio = 30.

**Figure 11:**
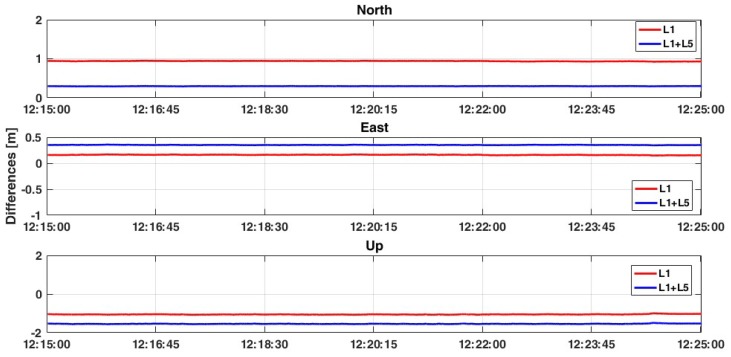
Comparison between L1 and L1+L5 solutions considering GPS+Galileo constellations and ratio = 30.

The test has continued introducing Galileo constellation which presents, for the GPS-only case, a better dual-frequency solution. In this case, the positioning estimation benefits from the new measurements (Table 8 and Figure 10). This behaviour is confirmed in the last test, where GLONASS measurements have been removed and the GPS+Galileo solution has been considered (Figure 11). For 2D positioning, less than 5 cm accuracy is achieved with a standard deviation of 1 cm. Observing the Up component, the best accuracy is obtained when all constellations are considered with the single frequency L1 (about 16 cm). Also RMS values are improved, especially for the planimetric components (Table 8): in this case, considering L1+L5 frequencies the RMS values are lower than 35 cm both for East and North components, even if the result for the up is worse than in the L1 case (1.526 m against 1.049 m).

All previous results are comparable with those obtained in many other sessions that are not presented in this paper. Thus, they can be considered independent by the sessions.

## 6. Conclusions

The precise positioning using mobile devices is still currently a challenge. The goal of cm-level accuracy in real-time has not yet been reached, mainly due to the quality of GNSS raw measurements obtained from smartphones. As discussed in previous studies, this is not the final purpose of the employment of these devices, especially because there are some unclear aspects, such as the difficulty of determining the exact position of the smartphone GNSS antenna. Even if the position of the GNSS chipset is known in most of the manufacturer schemas, the antenna position is usually not highlighted. So, the idea is to focus the attention on the precision of the solution, in order to guarantee high reliability of the obtained results.

From this study, it is confirmed that it is possible to reach a cm-level of precision if an RTK single-baseline positioning approach is considered, even if the master device is a smartphone too. Firstly, a typical RTK positioning using a geodetic receiver as master and a smartphone as rover is considered: this has represented the “best” possible scenario where a smartphone device can work in real-time, under the assumption that a CORS network is not available. In this case, this work has demonstrated once again that it is not useful to reach a fixed solution using smartphone devices, because both the quality of GNSS signals and the software available today for real-time positioning are not able to fix the phase ambiguities in the right way. Considering the FLOAT-only solutions, it is possible to reach a precision of about few mm both in the case of single- or multi-frequency solutions.

The exploitation of multi-constellation GNSS chipsets allows us to obtain better precision with respect to the GPS-only results, especially in when considering the smartphone-smartphone baseline: the best results can be obtained when considering the GPS+Galileo constellations, with a precision of about 2 cm both for 2D and Up components in the case of L1+L5 frequencies, or 3 cm for 2D components and 2 cm for the Up component in the case of an L1 frequency. When the GLONASS constellation is also added, the results are worse. In case of the employment of the only L1 frequency, the precision is about 13 cm for 2D components and 80 cm for the Up, while when adding the L5 frequency there is an improvement only for this last component, reaching a precision of about 50 cm. The accuracies are not bad too, allowing the achievement of a sub-meter solution for 2D components in all cases and a level of accuracy of about 1 m for the Up component.

In conclusion, it is possible to affirm that these results are very promising for cooperative positioning using smartphone devices in outdoor environments.

## Figures and Tables

**Table 1:** GNSS receivers used as master stations. *accuracy provided for illustrative purposes [14,18].

Characteristics	TORI CORS	Xiaomi Mi8
GNSS signals	GPS: C/A, L1, L2, L5, Doppler, S/N GLONASS: L1, L2 Galileo: E1, E5 BeiDou: B1, B2a	GPS: C/A, L1, L5, Doppler, S/N GLONASS: L1 Galileo: E1, E5 BeiDou: B1, B2a
Cost [€]	16000	800
Receiver	LEICA GRX1200+ GNSS	Broadcom BCM47755
Antenna	LEIAR25.R3 NONE	Microstrip patch antennas
Typical 3D positioning accuracy*	Less than 1 cm	≈ 10 m
Applications	CORS	location-based applications and services (LBS), navigation

**Table 2:** Main statistics parameters of collected signals from smartphone devices considering different GNSS constellations.

Satellite constellation	L1 Frequency	L5 Frequency
	Mean ± σ SNR values [dB-Hz]	Mean ± σ SNR values [dB-Hz]
**GPS**	38 ± 4	37 ± 2
**GLONASS**	37 ± 2	N/A
**Galileo**	36 ± 3	28 ± 2 *
**BeiDou**	30 ± 3	28 ± 4

* only 3 Galileo satellites have a SNR value on L5 frequency that is greater than 28 dB-Hz

**Table 3:** Case studies considered in this research activity.

	Master	Rover
**Case 1**	Geodetic receiver (CORS)	Smartphone
**Case 2**	Smartphone	Smartphone

**Table 4:** Comparison between FIX + FLOAT solutions with the FLOAT-only ones, if the L1 frequency is considered.

	L1 (FIX + FLOAT)			L1 (FLOAT)		
Component	Mean [m]	std [m]	RMS [m]	Mean [m]	std [m]	RMS [m]
East	−0.431	0.138	0.453	−0.401	0.002	0.401
North	−0.181	0.140	0.229	−0.186	0.001	0.186
Up	0.516	0.298	0.596	0.476	0.005	0.476

**Table 5:** Comparison of FIX+FLOAT solutions considering L1 and L1+L5 frequencies.

	L1			L1 + L5		
Component	Mean [m]	std [m]	RMS [m]	Mean [m]	std [m]	RMS [m]
East	−0.431	0.138	0.453	−0.409	0.029	0.230
North	−0.181	0.140	0.229	−0.23	0.010	0.186
Up	0.516	0.298	0.596	0.464	0.038	0.264

**Table 6:** Comparison between FIX+FLOAT solutions with the FLOAT-only ones, if the L1+L5 frequencies are considered.

	L1+L5 (FIX + FLOAT)			L1+L5 (FLOAT)		
Component	Mean [m]	std [m]	RMS [m]	Mean [m]	std [m]	RMS [m]
East	−0.409	0.029	0.230	−0.408	0.001	0.181
North	−0.23	0.010	0.186	−0.23	0.001	0.132
Up	0.464	0.038	0.264	0.462	0.004	0.191

**Table 7:** Positioning results considering the GPS-only constellation.

	L1			L1 + L5		
Component	Mean [m]	std [m]	RMS [m]	Mean [m]	std [m]	RMS [m]
East	−0.337	0.138	0.329	−0.532	0.011	0.408
North	1.132	0.031	1.147	0.790	0.013	0.230
Up	−1.397	0.192	1.386	−1.852	0.020	0.463

**Table 8:** Positioning results considering the GPS+GLONASS constellations. L1+L5 frequencies are used only for GPS constellation.

	L1			L1 + L5		
Component	Mean [m]	std [m]	RMS [m]	Mean [m]	std [m]	RMS [m]
East	−0.480	0.085	0.444	−0.656	0.120	0.539
North	1.045	0.071	1.015	0.807	0.048	1.372
Up	−0.744	0.296	0.923	−1.011	0.378	1.076

**Table 9:** Positioning results considering the GPS+GLONASS+Galileo constellations. L1+L5 frequencies are referred to only for GPS and Galileo constellations.

	L1			L1 + L5		
Component	Mean [m]	std [m]	RMS [m]	Mean [m]	std [m]	RMS [m]
East	−0.123	0.101	0.159	−0.027	0.135	0.138
North	0.976	0.128	0.984	0.421	0.070	0.427
Up	−0.167	0.809	0.825	0.386	0.506	0.637

**Table 10:** Positioning results considering the GPS+ Galileo constellations.

	L1			L1 + L5		
Component	Mean [m]	std [m]	RMS [m]	Mean [m]	std [m]	RMS [m]
East	0.131	0.011	0.132	0.332	0.008	0.332
North	0.931	0.033	0.932	0.298	0.015	0.298
Up	−1.049	0.017	1.049	−1.526	0.019	1.526
